# Metformin use in patients with type 2 diabetes mellitus is associated with reduced risk of deep vein thrombosis: a non-randomized, pair-matched cohort study

**DOI:** 10.1186/1471-2261-14-187

**Published:** 2014-12-15

**Authors:** Dai-Yin Lu, Chin-Chou Huang, Po-Hsun Huang, Chia-Min Chung, Shing-Jong Lin, Jaw-Wen Chen, Wan-Leong Chan, Hsin-Bang Leu

**Affiliations:** Division of Cardiology, Department of Medicine, Taipei Veterans General Hospital, Taipei, Taiwan; Department of Medical Research and Education, Taipei Veterans General Hospital, Taipei, Taiwan; Cardiovascular Research Center, Taipei Veterans General Hospital, Taipei, Taiwan; Institute of Pharmacology, Taipei Veterans General Hospital, Taipei, Taiwan; Institute of Clinical Medicine, National Yang-Ming University, Taipei, Taiwan; Institute of Biomedical Sciences, Academia Sinica, Taipei, Taiwan; Healthcare and Management Center, Division of Cardiology, Taipei Veterans General Hospital, 201 Sec. 2, Shih-Pai Road, Taipei, Taiwan

**Keywords:** Deep vein thrombosis, Metformin, Type 2 diabetes mellitus

## Abstract

**Background:**

Metformin, an insulin-sensitizer, may correct several physiologic abnormalities owing to insulin resistance in patients with type 2 diabetes mellitus (DM). The effects of metformin on venous thrombosis in patient with type 2 DM have not been reported. Our study strived to explore the relationship of metformin therapy and the subsequent development of deep vein thrombosis (DVT) using a nationwide, population-based database.

**Methods:**

From 1997 to 2003, we identified a study cohort consisting of patients with type 2 DM using metformin 7154 cases in the National Health Insurance Research Database. A control cohort without metformin, matched for age, sex, comorbidities, and medications was selected for comparison.

**Results:**

Of the 14945 patients (7167 patients with metformin vs. 7778 control), 60 (0.40%) patients developed DVT during a mean follow-up period of 3.74 years, including 16 (0.21%) from the cohort with metformin and 44 (0.56%) from the control group. Subjects with metformin experienced a 0.427 fold (95% confidence interval 0.240-0.758; *P* = 0.004) changes of risk reduction in development of DVT, which was independent of age, sex and co-morbidities. Kaplan-Meier analysis also revealed metformin therapy is associated with lower occurrence of DVT (log-rank test, *P* = 0.001).

**Conclusions:**

Metformin may have protective effect in patients with type 2 DM for DVT.

## Background

Vascular disease is a major cause of morbidity and mortality among patients with diabetes, and these patients account for a significant proportion of all patients with cardiovascular disease, including coronary artery disease, acute myocardial infarction, and cerebral infarction [1]. Insulin resistance contributes greatly to development of cardiovascular disease in patient with metabolic syndrome and type 2 diabetes mellitus (DM). Therefore, treatment with an insulin-sensitizing agent, such as metformin may correct several of the primary pathophysiologic abnormalities, including lipid metabolism, endothelial function, and platelet hyperactivity in patients with diabetes mellitus [2]. The United Kingdom Prospective Diabetes Study Group (UKPDS) has shown that patients with type 2 DM treated with metformin had a 36% lower risk of all-cause mortality and 39% lower risk of myocardial infarction respectively compared with those treated conventionally. This risk reduction was especially greater for metformin than insulin or sulphonylurea treatment, despite similar glycemic control [3]. Metformin appears to provide cardiovascular protection beyond blood sugar control. In addition to arterial vascular disease, pathology on venous system is also common in patients with diabetes. Epidemiological studies demonstrate an increased risk of deep vein thrombosis and pulmonary embolism among diabetic patients [4, 5]. Deep venous thrombosis frequently causes limitation on daily activities, while pulmonary embolism may further contribute to a life-threatening condition. The effects of metformin on venous thrombosis in patient with type 2 diabetes have not been previously reported. We hypothesized that metformin may reduce the development of venous thrombosis in patient with type 2 diabetes. Utilizing a nationwide database, we conducted this nonrandomized, pair-matched cohort study to investigate the relationship between metformin therapy and the subsequent development of deep venous thrombosis among patients with type 2 diabetes mellitus.

## Methods

### Database

The National Health Insurance program in Taiwan has been operating since 1995 and has enrolled nearly all the inhabitants of Taiwan (23,074,487 beneficiaries out of 23,162,123 inhabitants at the end of 2010). The National Health Insurance Research Database (NHIRD) at the National Health Research Institutes (NHRI) (http://w3.nhri.org.tw/nhird/en/index.htm) in Miao-Li (Taiwan) is in charge of the entire National Health Insurance claims database, and it has published numerous extracted datasets for researchers. The NHRI released a cohort dataset comprising 1,000,000 randomly sampled people who were alive during 1997 and collected all the records of these individuals from 1995 onwards. The database has been confirmed by NHRI to be representative of the Taiwanese population [6]. It is also one of the largest nationwide population-based databases in the world, with more than 1000 scientific articles published using its data [7]. In this cohort dataset, each patient’s original identification number has been encrypted to protect privacy. Of note, the encrypting procedure is consistent such that the linkage of claims belonging to the same patient is feasible within the NHIRD datasets. The current study was conducted using HNIRD dataset which contained patients’ all medical claim records, including coverage for outpatient, inpatient, emergency, dental, traditional Chinese medicine services, and prescription drugs. Because the NHIRD consists of de-identified secondary data released to the public for research purposes, this study was exempt from full review by the Institutional Review Board. The encrypting procedure is consistent, so the linkage of claims belonging to the same patient is feasible within the NHIRD.

### Study sample and control

We identified patients who used metformin for diabetes treatment (International Classification of Diseases, Ninth Revision, Clinical Modification [ICD-9-CM] codes 250.0 - 250.9) from the 1,000,000 sampling cohort dataset from January 1, 1997. An age-, sex-, and co-morbidity matched non-expose control group was selected from those patients who did not use metformin throughout the whole course of follow-up. To investigate the causal relationship between metformin use and deep venous thrombosis (DVT) occurrence, in both groups, subjects with pre-existing DVT (ICD-9-CM codes 453.0-453.9) before enrollment were excluded from this study. Women with diagnosis of pregnancy during study period were also excluded, since the first-line anti-hyperglycemic agent for gestational diabetes is insulin, not metformin [8]. The co-morbidities to be matched in the 2 groups included pre-existing (upon enrollment) hypertension (ICD-9-CM codes 401.xx–405.xx), coronary artery disease (CAD) (411.xx, 413.xx, 414.xx), hyperlipidemia (272–272.4). Previously well documented risk factors for DVT were recorded, such as cancer (140.xx-199.1), traumatic or pathologic fractures involving spine, pelvis, upper, and lower limbs (733.10-733.19, 800.xx-829.xx), and major cardiothoracic, abdominal, pelvic surgeries and orthopedic surgeries of lower limbs. Concurrent medications for blood sugar control, statin, anti-platelet, anti-coagulant agents or hormone replacement therapy were also recorded in our study.

### Deep vein thrombosis event measurement

The end point of the study was defined as occurrence of DVT (ICD-9-CM codes 453.0-453.9). In this database, the ICD codes of DVT and the drug code of metformin did not change throughout the whole follow-up period (1997–2003), assuring the consistency of the disease and medication registry. Similar coding to identify DVT events has also been also used in our previous study [9].

### Statistical analysis

Microsoft SQL Server 2005 (Microsoft Corporation, Redmond, Wash) was used for data management and computing. Statistical analyses were performed utilizing SPSS software (Version 18.0; SPSS, Inc., Chicago, Ill). All data were expressed as mean ± SD or percentage. Comparisons between the 2 groups were determined by independent Student’s *t* test for continuous variables or Pearson’s 2 test, Yates’ correction for continuity/Fisher’s exact test as appropriate for categorical variables. Survival analysis also was assessed using the Kaplan-Meier method, with the significance based on the log-rank test. Cox proportional hazards models were used to test the association of metformin use with DVT. Statistical significance was inferred at a 2-sided *P* value of <0.05.

## Results

**Figure 1:**
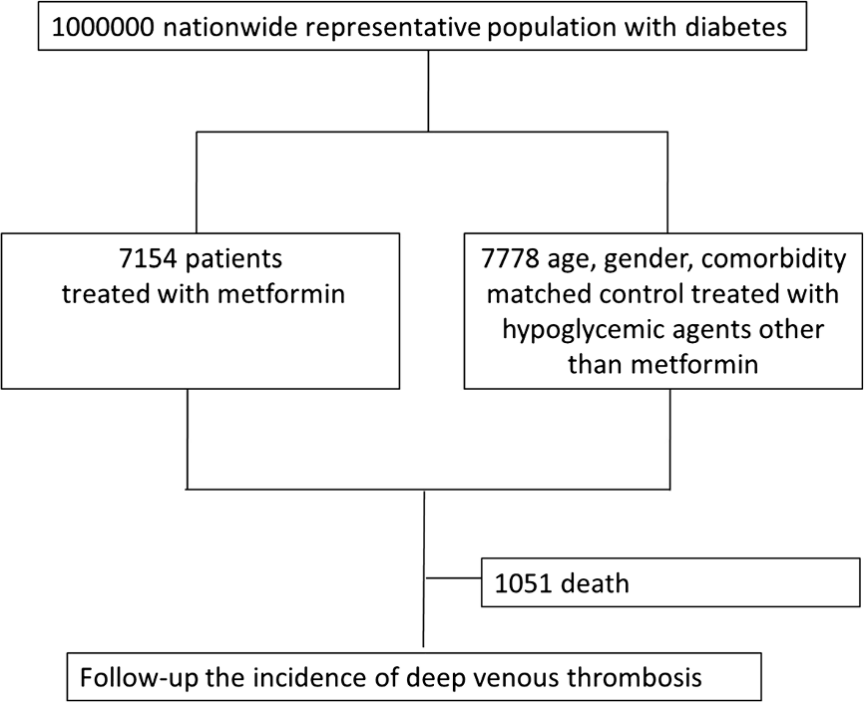
**Study flow chart.**

A total of 7167 type 2 DM patients who used metformin (mean age 57.70 ± 12.55 years) were identified from the 1,000,000 sampling cohort dataset between January 1997 and December 2003. Another 7778 subjects without metformin therapy (mean age 57.72 ± 13.37 years) were matched for age, sex, co-morbidities, medications, serving as the control group. The demographic parameters of study subjects are listed in Table 1. Figure 1 showed the entire flow of study and most enrollees in both groups (88.1% in metformin cohort and 88.6% in the control group) remained active and were followed through the end of the study at the end of the study period (December 31, 2003).

**Table 1:** **Baseline characteristics of the study population**

	Metformin		
	No (N = 7778)	Yes (N = 7167)	P value
Age, years	57.72 ± 13.37	57.70 ± 12.55	0.937
Male gender	4095 (52.6)	3808 (53.1)	0.565
Hypertension	1885 (24.2)	1695 (23.7)	0.413
CAD	671 (8.6)	535 (7.5)	0.010
Hyperlipidemia	905 (11.6)	849 (11.8)	0.708
Atrial fibrillation	69 (0.9)	35 (0.5)	0.005
Cancer	372 (3.8)	308 (4.3)	0.155
Fracture	538 (6.9)	364 (5.1)	<0.001
Major surgery	405 (5.2)	321 (4.5)	0.04
***Medications***			
Aspirin	696 (8.9)	692 (9.7)	0.144
Clopidogrel	12 (0.2)	8 (0.1)	0.625
Warfarin	31 (0.4)	36 (0.5)	0.409
Statin	263 (3.4)	342 (4.8)	<0.001
HRT	45 (0.6)	54 (0.8)	0.203
***Other anti-hyperglycemic agents***			
SU	7652 (98.4)	6941 (96.8)	<0.001
Meglitinide	2117 (27.2)	626 (8.7)	<0.001
α-glucosidase Inhibitor	2629 (33.8)	938 (13.1)	<0.001
TZD	2663 (34.2)	1188 (16.6)	<0.001
Insulin	5377 (69.1)	2342 (32.7)	<0.001
Kind of drug used	2.63 ± 0.84	2.68 ± 0.85	<0.001

Data are the mean ± SD and n (%).

CAD indicates coronary artery disease; HRT, hormone replacement therapy; SU, sulfonylurea; TZD, Thiazolidinediones.

Fracture includes traumatic or pathological fracture involving upper or lower limbs, spine and pelvis.

Major surgery includes cardiothoracic, abdominal, pelvic surgery or orthopedic surgery of lower limbs.

**Figure 2:**
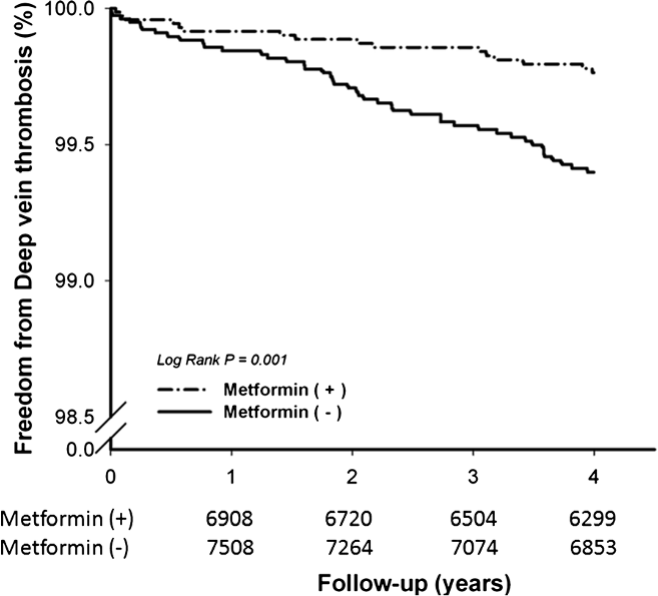
**Kaplan-Meier curves revealed patient under metformin therapy was associated with lower incidence of deep vein thrombosis (log-rank test, p = 0.001).**

During an average of 3.74 ± 0.80 years’ follow-up period, there was a significantly lower incidence of DVT development among patients with type 2 diabetes who used metformin, compared with the control group (16 [0.22%] *vs* 44 [0.56%], *P* < 0.001). Figure 2 outlines the results of a Kaplan-Meier analysis and the log-rank test, which showed that metformin therapy was associated with a significantly lower incidence of DVT than those without metformin (Log-rank *P* = 0.001). The significant difference between groups was observed in the first year, even just after six months treatment of metformin. Comparison between patients with and without DVT was shown in Table 2. Patients with DVT were older, more likely to be male, with more co-morbidities of coronary artery disease, fractures and performed more major surgeries.

**Table 2:** **Baseline characteristics of the DVT population**

	DVT		
	No (N = 14885)	Yes (N = 60)	P value
Age, years	57.69 ± 12.98	62.35 ± 13.31	0.006
Male gender	7867 (52.8)	36 (60)	0.328
Hypertension	3560 (23.9)	20 (33.3)	0.120
CAD	1194 (8.0)	12 (20)	0.003
Hyperlipidemia	1745 (11.7)	9 (15)	0.558
Atrial fibrillation	102 (0.7)	2 (3.3)	0.065
Cancer	674 (4.5)	680 (4.6)	0.054
Fracture	894 (6.0)	8 (13.3)	0.027
Major surgery	716 (4.8)	10 (16.7)	0.001
***Medications***			
Aspirin	1381 (9.3)	7 (11.7)	0.679
Clopidogrel	20 (0.1)	0 (0.0)	1.000
Warfarin	63 (0.4)	4 (6.7)	<0.001
Statin	601 (4.0)	4 (6.7)	0.308
HRT	99 (0.7)	0 (0.0)	1.000
Metformin	7151 (48.0)	16 (26.7)	0.001

Data are the mean ± SD and n (%).

CAD indicates coronary artery disease; HRT, hormone replacement therapy.

Fracture includes traumatic or pathological fracture involving upper or lower limbs, spine and pelvis.

Major surgery includes cardiothoracic, abdominal, pelvic surgery or orthopedic surgery of lower limbs.

The Cox proportional hazards regression model was used to determine the factors independently associated with the development of DVT. After adjusting for age, sex, and the aforementioned significant co-morbidities, only age (hazard ratio [HR] 1.031; 95% CI, 1.009-1.053); *P* = 0.004), CAD (HR 2.208; 95%, CI, 1.026-4.751; *P* = 0.043), and major surgery ([HR] 3.161; 95% CI, 1.535-6.510; *P* = 0.002) were independently associated with DVT development, while metformin was independently associated with lower risk of DVT (HR 0.427; 95%, CI, 0.240-0.758; *P* = 0.004) (Table 3).

**Table 3:** **Predictors of deep vein thrombosis identified by uni- and multi-variate Cox regression analysis**

	Univariate analysis		Multi-variate analysis	
	HR (95% CI)	P	HR (95% CI)	P
Age, per year	1.032 (1.010-1.054)	0.004	1.031 (1.009-1.053)	0.004
Male gender	1.361 (0.812-2.282)	0.242	1.577 (0.934-2.661)	0.088
Hypertension	1.611 (0.942-2.755)	0.082	1.007 (0.541-1.874)	0.983
CAD	2.956 (1.570-5.564)	0.001	2.208 (1.026-4.751)	0.043
Hyperlipidemia	1.290 (0.635-2.621)	0.481	0.921 (0.407-2.084)	0.844
Cancer	2.694 (1.159-6.263)	0.021	1.598 (0.657-3.888)	0.301
Fracture	2.495 (1.185-5.251)	0.016	1.918 (0.899-4.094)	0.092
Major surgery	4.055 (2.057-7.996)	<0.001	3.161 (1.535-6.510)	0.002
Aspirin	1.315 (0.598-2.891)	0.496	0.703 (0.291-1.701)	0.435
Dipyridamole	1.014 (0.368-2.797)	0.978	0.728 (0.257-2.063)	0.550
Statin	1.667 (0.604-4.596)	0.324	1.553 (0.499-4.836)	0.448
Metformin	0.395 (0.223-0.700)	0.001	0.427 (0.240.0.758)	0.004

CAD indicates coronary artery disease.

Fracture includes traumatic or pathological fracture involving upper or lower limbs, spine and pelvis.

Major surgery includes cardiothoracic, abdominal, pelvic surgery or orthopedic surgery of lower limbs.

## Discussions

Our major findings of the present study demonstrated metformin use was associated with reduced risk of developing DVT among patients with type 2 DM using a large-scale nationwide database in Asian population. In addition, increasing age and history of cardiovascular disease were found to contribute independently to the risk of DVT.

Metformin is an insulin-sensitizing biguanide used to treat type 2 DM. The glucose-lowering effect is a consequence of reduced hepatic gluconeogenesis and increased insulin-stimulated glucose uptake in skeletal muscle and adipocytes [10, 11]. In addition to the anti-hyperglycemic effects, metformin provides additional cardioprotective effects beyond sugar lowering, which may be related to the actions of metformin on lipid metabolism, vascular smooth muscle and cardiomyocyte intracellular calcium handling, endothelial function, hyper-coagulation and platelet hyperactivity. It also provide greater protection against the development of macrovascular complications than would be expected from its effects on glycemic control alone [12]. There are statistically significant reductions in the risk of all-cause mortality and diabetes-related mortality [3]. Either as monotherapy or in combination with sulfonylurea, metformin was associated with reduced all-cause and cardiovascular mortality compared with sulfonylurea monotherapy [13]. The UKPDS post-trial reported significant and persistent risks reductions for diabetes-related end point, myocardial infarction and death from any cause [14]. Current guidelines from the American Diabetes Association/European Association for the study of Diabetes (ADA/EASD) recommend early initiation of metformin as a first-line drug for monotherapy and for combination therapy for patients with type 2 diabetes [15].

Our cohort study is the first study to document the protective effect of metformin on venous thromboembolism using a large-scale nationwide cohort database. It is widely accepted that DM impairs endothelial nitric oxide synthase (eNOS) activity and enhances the production of reactive oxygen species (ROS), thus resulting in diminished NO bioavailability and the consequent pro-atherogenetic alterations [16]. Insulin is a normal regulator of eNOS activation and NO production through successive phosphorylation. Insulin resistance in DM attenuates the process and suppresses the normal NO secretion [17]. In previous studies, metformin treatment significantly improved glycation, oxidative stress, nitric oxide (NO) bioavailability and insulin resistance and normalized endothelial function in aorta of rats with diabetes [18]. In human, subjects who received metformin had improvement in endothelium-dependent, acetylcholine-stimulated flows compared with those treated with placebo. In young women with polycystic ovary syndrome, which have an increased prevalence of insulin resistance, short-term metformin therapy improves arterial stiffness and endothelial function [19]. Although the mechanism was still not clear, metformin may help restore endothelial function via modulation of insulin resistance.

It has been reported that metformin treatment was associated with improvements in plasma markers of endothelial function, including von Willebrand factor (vWF), soluble vascular adhesion molecule-1 (sVCAM-1), tissue-type plasminogen activator (t-PA), plasminogen activator inhibitor-1 (PAI-1) and soluble intercellular adhesion molecule-1 (sICAM-1), suggesting the benefit of endothelial function improvement and inflammation reduction [20]. Metformin have specific effects on endothelial function protection, which explained about 34% of the reduction in the in the risk of CV morbidity and mortality [20]. These findings indicate that metformin is able to improve endothelial reactivity at the macro- and microcirculatory level, both of which relate to cardiovascular outcomes [21, 22].

Insulin resistance is associated with hypofibrinolysis, and metformin has been shown to improve insulin sensitivity and fibrinolysis [23]. In animal model, metformin prolonged activated partial thromboplastin and prothrombin times, and the endothelial cell damage improved [24]. Sobel et al. demonstrated insulin-sensitizing strategy led to lower fibrinogen level [25]. It may alter fibrin structure and function by interfering with the process involved in fibrin polymerization and lateral aggregation [26]. A reduction in coagulation factor VII levels and factor VIII activity had also been demonstrated [26, 27]. In subjects with obesity, there was a significantly greater decrease in tissue plasminogen activator (t-PA) antigen and vWF in the metformin than in the placebo group. The use of metformin increased t-PA activity and decreased t-PA antigen in patients with insulin resistance and hypertension [23, 28]. Patients with newly diagnosed type 2 diabetes treated with 8-week metformin received an intravenous infusion of L-arginine before and after metformin treatment, while L-arginine is the natural precursor of NO and may be useful to assess endothelium-dependent vascular function in humans. The decrease in both platelet aggregation and blood viscosity after L-arginine was significantly amplified after metformin [29]. Therefore insulin sensitizing therapy with metformin may alter the coagulation profiles, which bring beneficial consequences in the thrombogenesis. Together all, metformin may have better endothelial protection and coagulation, which may provide possible mechanisms connected to the reduced DVT events observed in our study.

A number of published studies have shown that the incidence of first-time venous thromboembolism (VTE) rises with age. The incidence increases dramatically after age 60 [30, 31]. Although use of oral contraceptives and post-menopausal hormone replacement have been associated with VTE in women, published data suggest no consistent differences in the incidence of VTE among men and women [31]. Besides, we demonstrated that history of cardiovascular disease is associated with development of DVT. In recent years, epidemiological studies have explored the association between VTE, arterial thromboembolism (CVD, MI, stroke) and atherosclerosis, indicating patients with DVT had an increased relative risk for MI and stroke [32]. Since they share several common risk factors, including smoking, immobility, and as our patient group – DM, it is not surprising that a patient with CVD may have increased risk of venous thromboembolism [33].

Previous studies reported metformin treatment was associated with a decrease in vitamin B12 concentration [34], which was present in 5.8% of the population [35] and vitamin B12 deficiency may be associated with hyperhomocysterinemia. However the association between vitamin B12 deficiency and deep vein thrombosis remained undetermined. Previous data have suggested that there is no adequate evidence concerning the role of metformin therapy and hyperhomocysteinemia. A previous study from Thailand demonstrated s that although metformin may have caused low vitamin B12 levels, there were no significant changes to homocysteine levels [36]. Hoogeveen et al. further found that metformin-exposed patients had only slightly higher serum total homocysteine levels then control group [37, 38]. Furthermore, the dose–response relationship between cumulative exposure to metformin and total homocysteine level was not identified [38]. Besides medications, other dietary factors such as fruit and vegetable consumption in diabetic patients could also be strong independent determinants of homocysteine levels [39]. Recent studies show that lowering homocysteine levels does not decrease the risk for atherosclerosis or thrombosis [40, 41]. This supports the theory that homocysteine may just be an “innocent bystander” and not the cause of these conditions. Therefore, large-scale prevention studies identifying high-risk patients through genetic tests, like C677T homozygous mutation targeting on populations with low folate intake have not been performed [42].

In addition, it has been reported that serum vitamin B12 levels do not adequately assess tissue vitamin B12 stores [43]. Patients with type 2 diabetes may show normal extracellular vitamin B12, but disturbed intracellular B12-dependent biochemical reactions. Metformin treatment was associated with low serum vitamin B12 level, while improved intracellular vitamin B12 metabolism despite low serum vitamin B12 [44].

There are some limitations in our study. First, it is a nonrandomized, pair-matched cohort study. The diagnoses of type 2 DM and DVT rely on ICD coding from insurance claim database. The diagnosis of deep venous thrombosis is confirmed by venography or lower limb Doppler ultrasonography, while the arrangement and interpretation of these image studies depend on clinician’s judgment. Therefore the incidence of deep venous thrombosis may be underestimated. In addition, some personal information, including smoking, immobilization or concurrent medication was not available in the administrative data. Since smoking, immobility, or use of contraceptive agents are well recognized risk factors of deep vein thrombosis [45]. Accurate assessment of the contributory and confounding effect of these factors are not feasible. Because of the limitation of National Health Insurance Research Database (NHIRD), we were unable to calculate the propensity score as correction of comorbidities. Individual biochemistry data was not available in this population-based registry. Therefore, we cannot measure patients’ coagulation and inflammation profiles, and the relationship cannot be delineated. Despite these limitations, this study was believed to give the first insight about the effect of metformin on venous thromboembolism.

## Conclusion

In conclusion, we identified that metformin therapy may be associated with a protective effect in patients with type 2 diabetes mellitus from deep vein thrombosis in a large-scale population-based study. Further larger prospective studies or meta-analysis are needed to confirm our findings.

## Abbreviations

CVD: Cardiovascular disease
DM: Diabetes mellitus
DVT: Deep vein thrombosis
eNOS: Endothelial nitric oxide synthase
ICD: International classification of diseases
NHRI: National Health Research Institutes
NO: Nitric oxide
PAI-1: Plasminogen activator inhibitor-1
PE: Pulmonary embolism
ROS: Reactive oxygen species
sICM-1: Intercellular adhesion molecule-1
sVCAM-1: Soluble vascular adhesion molecule-1
t-PA: Tissue-type plasminogen activator
VTE: Venous thromboembolism
vWF: von Willebrand factor.
